# The effects of aerobic training before and after the induction of Alzheimer’s disease on ABCA1 and APOE mRNA expression and the level of soluble Aβ1-42 in the hippocampus of male Wistar rats

**DOI:** 10.22038/ijbms.2018.32911.7860

**Published:** 2019-04

**Authors:** Zahra Sarlak, Mahtab Moazzami, Moazzami Attarzadeh Hosseini, Reza Gharakhanlou

**Affiliations:** 1 Faculty of Sport Sciences, Ferdowsi University of Mashhad, Mashhad, Iran; 2 Faculty of Humanities, Tarbiat Modares University, Tehran, Iran

**Keywords:** ABCA1, Aerobic training, Alzheimer’s disease, APOE, Soluble Aβ1-42

## Abstract

**Objective(s):**

The purpose of this study was to investigate the effects of aerobic training before and after the induction of Alzheimer’s disease on ABCA1 and APOE mRNA expression and the level of soluble Aβ1-42 in the hippocampus of male Wistar rats.

**Materials and Methods:**

Ninety six eight-week-old male Wistar rats were randomly divided into two groups: Training (n=48) and Rest (n=48). After four weeks, each group was randomly divided into two subgroups: intra-hippocampal injection of Aβ1-42 (n=24) and DMSO (n=24). Then, each group was again randomly divided into two groups: Training (n=12) and Rest (n=12). After four weeks, each group was again randomly divided into two groups: Behavioral test (n=7) and sacrificed (n=5).

**Results:**

The one-way ANOVA showed a significant increase in the mRNA expression of ABCA1 (*P*<0.05), a significant decrease in the level of soluble Aβ1-42, and no significant difference in the expression of APOE mRNA (*P*>0.05) in the hippocampus as a result of training. The analysis of the Morris water maze data showed that intra-hippocampal injection of Aβ1-42 impaired spatial learning and memory and exercise improved spatial learning (*P*<0.05) and memory (*P*<0.05).

**Conclusion:**

Therefore, aerobic training by a significant increase in the mRNA expression of ABCA1, which is the main factors of lipid metabolism in the brain and which is involved in the pathology of Alzheimer’s disease, can be consistent with improving cognitive function as an effective way of preventing and improving the symptoms of Alzheimer’s disease.

## Introduction

Alzheimer’s is a progressive neurodegenerative disease and the most common form of dementia (1). Alzheimer’s disease (AD) is associated with the atrophy of the hippocampus and the cerebral cortex (2). It begins with short-term memory impairment and results in the complete decline of cognitive function (3, 4). AD is clinically diagnosed by progressive cognitive im

pairment (5) but pathologically diagnosed by Neurofibrillary Tangles (NFTs) and extracellular amyloid plaques. NFTs include tau hyper-phosphorylated proteins in the brain or the hippocampal cortex (2), and the amyloid plaques are mainly caused by the fibrillation of beta-amyloid peptide (Aβ) (6-8). The level of Aβ in the brain is determined by the dynamic balance between the factors that produce it from the protein of the amyloid precursor (APP), which is a transmembrane protein, and the factors that clear it from the brain. Thus, the deficiency of the balance between the production and the clearance of Aβ can cause the accumulation and deposition of the Aβ peptide in the brain of AD patients, and the factors that stimulate the cleavage of APP or reduce the clearance of Aβ peptide are considered as the risk factors of AD (9).

Recent research has shown a link between lipid metabolism and AD and has identified some risk factors of the early onset of AD related to cholesterol metabolism (10-12). Lipid metabolism in the brain is widely differentiated from its peripheral metabolism due to the blood-brain barrier and different lipoproteins. Thus, identifying the mechanism of lipid metabolism in the brain may be a new therapeutic strategy in AD (13). Therefore, some factors are involved in the metabolism of cholesterol and lipids in the brain. Two of these are apolipoprotein E (APOE) and ABCA1 (ATP-binding cassette transporter), whose role in AD pathology, as recent studies consider, is to influence the production and clearance of Aβ.

APOE is the most important apolipoprotein in the brain, mainly synthesized by astrocytes and a little by microglia (14). It is a chaperone for Aβ; it affects the deposition and clearance of Aβ (15, 16). APOE increases the proteolytic degradation of Aβ by NEP (Neprilysin) and IDE (Insulin Degrading Enzyme), especially when it is lipidated (14). On the other hand, the clearance of Aβ1-42 is less dependent on the efflux from the blood-brain barrier, and the role of proteolytic clearance by NEP and IDE is greater (17). Therefore, the levels of APOE and lipidated APOE affect Aβ metabolism (14, 18).

ABCA1 is a membrane protein that mediates the cholesterol efflux to low-lipid or lipid-free apolipoproteins. ABCA1 transports the cholesterol to the APOA1 in the periphery and to the APOE in the brain (19, 20). The role of ABCA1 in regulating plasma HDL (high-density lipoprotein) levels in atherosclerosis has been considered. In addition, due to its effects on lipidization and APOE stability, it is involved in AD pathology. ABCA1 increases the degradation of Aβ by increasing the lipidation of APOE and can cause lower Aβ deposition and higher clearance of Aβ (21). On the other hand, APP is embedded in the cell membrane and cleaved by α-secretarase and β-sucrose enzymes that exist in the membrane (22, 23). The increased intracellular or membrane cholesterol can lead to the increasing cleavage of APP and produce Aβ. Therefore, ABCA1 can decrease Aβ production through the transport of cholesterol to APOE (23). As a result, the increase in the expression of ABCA1 by a decrease in the production of Aβ, and on the other hand, by an increase in the clearance of Aβ, is associated with preventing and treating AD.

Most studies have shown that physical activity and exercise can be effective in improving memory, learning and cognitive function (24-27) and possibly in reducing the risk of dementia (28). In addition, researchers have suggested that the cholesterol metabolism disorder in the brain is involved in the pathogenesis of AD (14), and the polymorphism of genes that regulate the cholesterol metabolism, such as ABCA1 and APOE, increase the risk of AD (23). But the effects of exercise on lipid metabolism in the brain and its role in preventing and improving the symptoms of AD have not been studied. Studies in this regard have been carried out using genetic and pharmaceutical interventions. Both in vivo and in vitro studies have shown that the down-regulation of the ABCA1 expression reduces the APOE in the brain, the cerebrospinal fluid, and plasma, and increases the Aβ load of the brain (29-33), while the up-regulation of the ABCA1 expression leads to an increase in the lipidation of APOE and reduces the Aβ load (34). Studies have also indicated that the administration of the RXRs (Retinoid X Receptors) and LXRs (Liver X Receptors) agonist increases the ABCA1 and APOE expressions, leads to an increase in the clearance of Aβ by lipidated APOE and causes an improvement in cognitive impairment (34-37). Therefore, because of the strong link between lipid metabolism in the brain and AD and since no studies have been done to investigate the effect of exercise, the purpose of this study was to investigate the effects of aerobic training before and after the induction of AD on ABCA1 and the APOE mRNA expression and the level of soluble Aβ1-42 in the hippocampus of male Wistar rats.

## Materials and Methods

***Animals***

In this experimental study, 96 adult male Wistar rats (8 weeks old, 20 ± 195 g weight) were used. Five rats were housed in a Plexiglas cage in a 12-hr light/dark cycle. The temperature was 22 ± 3 ^°^C and humidity was about 45%. There was no restriction on the animals’ access to water and food. After a week of familiarization with the environment, all rats were exposed to a familiarization with the treadmill for a week (10 min, speed: 10 m/min, 5 days). Then, the rats were randomly classified into a training group (48 rats) and a resting group (48 rats). After four weeks, each group was randomly classified into two subgroups: The Aβ group (24 rats injected with Aβ in the hippocampus) and the sham group (24 rats injected with DMSO in the hippocampus). After seven days, each of these groups was randomly classified into two subgroups: Subgroup 1 (out of 12 rats, which had undergone endurance training for four weeks and 48 hr had passed after their last training session, five were sacrificed and seven took a behavioral test), and Subgroup 2 (out of 12 rats, which had not undergone endurance training and resting for four weeks, five were sacrificed simultaneously with the training group and seven took the behavioral test. All experiments were done following the guidelines of United States National Institutes of Health (NIH) Guide for the Care and Use of Laboratory Animals and were approved by the Ethics Committee on the use of animals of Ferdowsi University of Mashhad, Mashhad, Iran (code of approval: IR.MUM.FUM.REC.1396.06).

***Exercise training protocol***

The aerobic exercise protocol was performed on a treadmill with a gradient of zero degrees five days a week for four weeks in both pre- and post-conditioning phases. This protocol was mild to moderate and the speed and duration were gradually increased (Table 1). The exercise-group rats were monitored during the training sessions and were encouraged by a weak electrical shock (0.5 mA intensity) that did not cause stress in the animal (38, 39).

***Preparation of Aβ1–42 & stereotaxic***

We utilized the intra-hippocampal injection of Aβ1–42 peptide to generate the AD model. To prepare the Aβ1-42 peptide, Aβ1-42 (Abcam, USA) was dissolved in a buffer solution (DMSO 3%) (Sigma Aldrich, USA) at the rate of 5 μg/μl. Then, they were divided into 30 μl per vial and kept at -80 ^°^C. The amyloid solution was incubated for seven days at 37 ^°^C to fibrillate the Aβ1–42 peptides (40). Then, all the animals were anesthetized with the intraperitoneal injection of ketamine (100 mg/kg) and xylazine (10 mg/kg) and they underwent stereotaxic surgery according to the Atlas of Paxinus and Watson: Anterior-posterior (AP), 3.8 mm; medial-lateral (ML), ±2.2 mm from the central sagittal line and dorsal-ventral (DV), -2.7 mm to the surface of the skull (41). In the Aβ groups, the Aβ1-42 peptide (1 μl) was injected into the CA1 area of the right and left hippocampus by the Hamilton syringe, and in the sham groups, the DMSO buffer (1 μl) was injected into the CA1 area of each hippocampus. 

***Tissue biopsies***

Twenty-four hours after the last training session, the rats were anesthetized by intraperitoneal injection of ketamine (100 mg/kg) and xylazine (10 mg/kg). Then, the animals were decapitated and the brain was removed from the skull bone. Subsequently, the hippocampal tissue was separated from the rest of the brain tissue, frozen with liquid nitrogen and stored at -80 ^°^C for future analysis. 

**Table1:** Exercise training protocol

	**Set ** *time (min)	**Speed (m/min)**	**Days**
**Week 1**	15*2	10	5
**Week 2**	15*2	10	5
**week3**	15*3	15	5
**Week 4**	15*4	15	5

**Table 2:** Oligonucleotide primer sequences

**Gene**	**Forward and reverse primer sequences **(5' → 3')	**Melting temperature (Tm)**	**Product size (bp)**
**ABCA**_1_	F: TCATGTACCCAGCGTCCTTT R: CCACACTACCATTGATGCCG	C° 82.74	96
**APOE**	F: ATCTGTCACCTCCTGCTCT R: CTTTTCCTTCCGCTGACTGG	C° 79.46	116
**GAPDH**	F: AAG TTC AAC GGC ACA GTC AAG G R: CAT ACT CAG CAC CAGCAT CAC C	C° 82.75	121

**Table 3:** The APOE and ABCA1 expressions and the level of soluble Aβ1-42 in the hippocampus of male Wistar rats in the eight groups. Each column is for each group including five rats

**Eta**	**P**		**F**	**TAT**	**TDT**	**TAR**	**TDR**	**RAT**	**RDT**	**RAR**	**RDR**		
**0.73**	0.032		2.576	5.9344	7.2283	2.8837	3.5871	4.2941	6.3204	1.2059	1	Mean	**ABCA1**
				4.4343	4.8307	0.7554	3.5228	3.0281	4.1496	1.2509	0	Std.	
**0.012**	1.000	0.075		1.0668	1.0409	0.9243	1.0891	0.9944	0.8470	0.9847	1	Mean	**APOE**
				1.2624	0.5557	0.8117	1.1225	0.5066	0.3383	0.3819	0	Std.	
**0.967**	0.000	134.4		26.2980	15.7300	35.2200	17.2820	31.7600	15.5000	46.0800	17.2060	Mean	**Aβ1-42**
				1.7919	0.8303	2.8083	2.4055	2.7553	1.2549	1.7922	2.8055	Std.	

Data expressed as Mean ± SD

**Table 4:** The escape latency, traveled distance, and time spent on the target quadrant (probe trial) of male Wistar rats in the eight groups. Each column is for each group including seven rats

**Eta**	**P**		**F**		**TAT**		**TDT**		**TAR**		**TDR**		**RAT**		**RDT**		**RAR**		**RDR**				
**0.816**	0.000		30.32		854.74		658.85		891.29		842.77		891.39		738.00		1069.5		864.99		Mean		**Travel distance**
					45.78		72.81		59.14		60.27		64.59		56.09		26.94		63.74		Std.		
**0.663**	0.000	13.50		37.35		26.95		39.04		36.37		38.38		28.66		47.67		36.36		Mean		**Escape latency**	
				4.10		5.20		4.75		4.61		5.25		4.47		5.06		3.14		Std.			
**0.650**	0.000	12.75		24.11		33.52		22.91		25.03		23.31		32.57		17.00		24.01		Mean		**Probe**	
				4.07		4.84		3.15		4.08		3.66		4.02		4.43		3.35		Std.			

Data expressed as Mean ± SD

**Figure 1:**
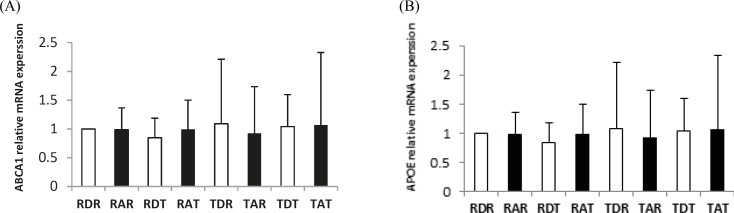
Real-Time PCR of hippocampus ABCA1 (A) and APOE (B) relative mRNA expression in eight groups .Rest-DMSO-Rest (RDR), Rest- Aβ -Rest (RAR), Rest-DMSO-Training (RDT), Rest-Aβ-Training (RAT), Training-DMSO-Rest (TDR), Training- Aβ -Rest (TAR), Training- DMSO - Training (TDT), Training- Aβ - Training (TAT)

**Figure 2:**
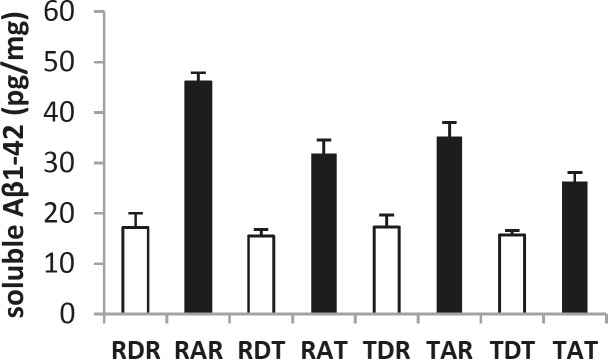
The level of soluble Aβ1-42 in the hippocampus in eight groups. Rest-DMSO-Rest (RDR), Rest- Aβ -Rest (RAR), Rest-DMSO-Training (RDT), Rest-Aβ-Training (RAT), Training-DMSO-Rest (TDR), Training- Aβ -Rest (TAR), Training- DMSO - Training (TDT), Training- Aβ - Training (TAT)

**Figure 3:**
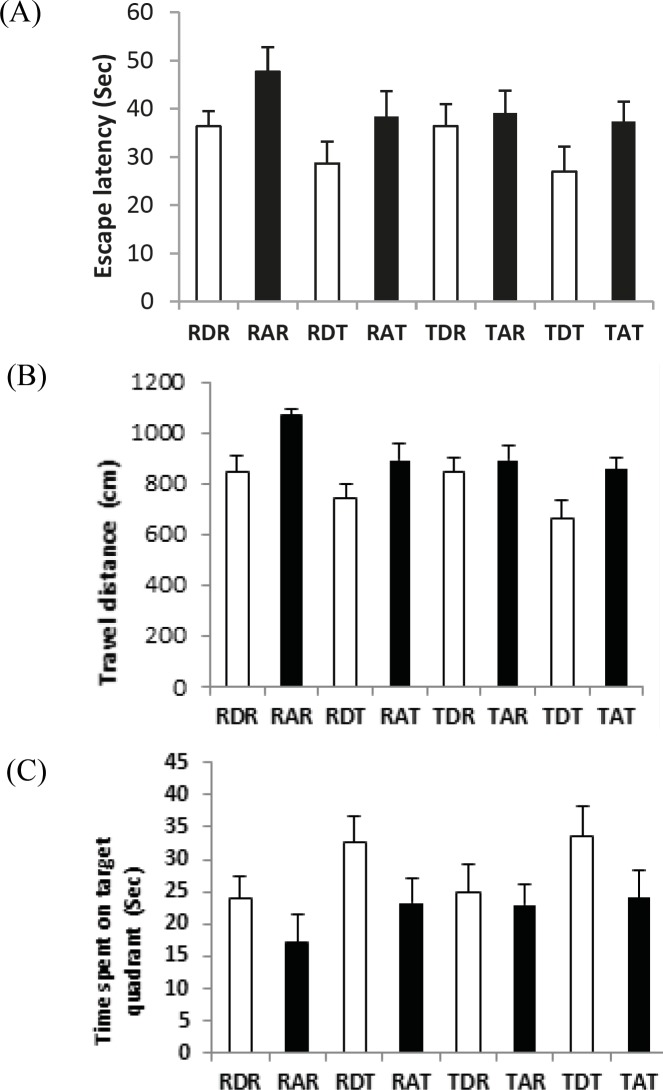
The escape latency (A), the travel distance (B) and the time spent on the target quadrant (C) in Morris water maze in eight groups .. Rest-DMSO-Rest (RDR), Rest- Aβ -Rest (RAR), Rest-DMSO-Training (RDT), Rest-Aβ-Training (RAT), Training-DMSO-Rest (TDR), Training- Aβ -Rest (TAR), Training- DMSO - Training (TDT), Training- Aβ - Training (TAT)

**Figure 4:**
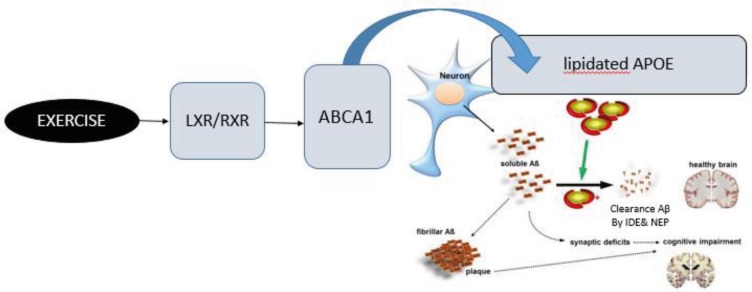
The proposed mechanism through which exercise can improve the clearance of Aβ. Exercise likely as an LXR/RXR agonist increased mRNA expression ABCA1 and subsequently, leading to an increase in lipidation of APOE. The lipidated APOE facilitates the proteolytic degradation of Aβ by NEP and IDE and promotes the clearance of Aβ

***RNA extraction, cDNA synthesis, and real-time PCR***

The total RNA was extracted from the right hippocampus tissue homogenized in TRIzol solution (1 ml) according to the manufacturer’s instructions. The concentration of RNA was determined by the Nanodrop spectrophotometer and 1000 ng RNA was used to synthesize cDNA. The cDNA was synthesized by a high-capacity cDNA reverse transcription kit (Applied Biosystems) following the instructions of the cDNA synthesis kits. Real-time PCR was performed using the RealQ Plus 2x Master Mix Green (AMPLIQON) and using 1 µl cDNA, 10 µl Master Mix, 1 µl of each of the forward and reverse primers, and 7 µl RNase-free water. All PCR reactions were performed in duplicate. The polymerase chain reaction was performed in the following thermal conditions: denaturation at 95 °C for 10 min, followed by 40 cycles of 95 °C for 15 sec, and annealing at 60 °C for 30 sec. The oligonucleotide primer sequences for ABCA1, APOE, and GAPDH (as an internal control) genes are listed in Table 2. The mRNA expression relative values were analyzed by the 2^–ΔΔCt^ method (42).

***ELISA***

Left hippocampus tissue of rats was homogenized with 1 ml of PBS solution and kept at -20 °C for one night. After applying two freeze-thaw cycles to break down the cell membranes, the homogenized tissue was centrifuged at 5,000 rpm for five min and the supernatant was extracted by the sampler. Subsequently, the hippocampal samples were used to determine the levels of soluble Aβ1-42 using the ELISA kit (CSB-E10786r; Cusabio Biotech, China), based on its manufacturer’s protocol. 

***Behavioral test***

The Morris water maze test was used to measure memory and learning. We used a dark circular pool with a diameter of 150 cm and a depth of 60 cm. The pool was filled with water (21 ^°^C ± 2) to a depth of 30 cm. A hidden platform with a 10 cm diameter was located at the center of the southeast (SE) quadrant, about 2 cm below the water surface. The escape latency and the traveled distance and time in each quadrant were recorded by a video camera placed above the pool and was analyzed using the Ethovision Video Tracking System. The Morris water maze protocol was as follows:

A) Learning stage: At this stage, the rats were trained for four consecutive days, each day in four separate trials, to find the hidden platform. At the start of each trial, each rat was placed on the platform for 15 to 20 sec. Then, the animal was randomly placed in the water from one of the four starting points (north, south, east, and west). The animal was allowed to swim and find the hidden platform located in the SE quadrant. If the rat could not find the platform within 60 sec, it would be guided by hand. Once it found the platform, the animal was allowed to remain on it for 20 sec. The escape latency and traveled distance were measured and recorded in each trial. 

B) Probe trial: A day after the learning stage, the spatial memory of the animals was evaluated in the probe trial. In this stage, the rats were evaluated in a 60-second test in which the Plexiglas platform was removed from the water. The time spent in the target quadrant was recorded (43).

***Statistical analysis***

To determine the normality of the distribution, we used the Shapiro-Wilk test. Also, Levine’s test was used to check the homogeneity of the variances. The Multivariate analysis of variance (MANOVA) and one-way ANOVA with Tukey’s *post hoc* test were used for the statistical analysis of the data at the significant level (*P*≤0.05). All results are expressed as mean ± SEM. 

## Results

***Analysis of biochemical parameters***

In this research, 40 male Wistar rats were sacrificed and the APOE and ABCA1 expression and the level of soluble Aβ1-42 in the hippocampus were measured in eight groups. Figures 1 and 2 show changes in the level of soluble Aβ1-42, and APOE and ABCA1 expression in the hippocampus of the eight groups. The results of the MANOVA test showed a significant difference between the eight groups with respect to these three dependent variables: Wilks ˄ = 0.020, F (21, 86.7) = 12.070, *P* = 0.000, Eta = 0.730, indicating that aerobic training has a significant effect on the APOE and ABCA1 expressions and the level of soluble Aβ1-42 in the hippocampus of rats (*P*<0.01), and 73% of the variability of these three variables was influenced by this training program. Table 3 shows the results by a one-way ANOVA for comparing APOE and ABCA1 expressions, and the level of soluble Aβ1-42 in the hippocampus of the rats of these eight groups.

The results of Table 3 show that aerobic training can significantly change the ABCA1 expression (*P*<0.05) in the hippocampus of rats. However, changes in the expression of the APOE gene were non-significant (*P*>0.05). The results also showed that aerobic training can significantly change the level of soluble Aβ1-42 in the hippocampus of rats (*P*<0.01).

The results of the *post hoc* test showed a significant difference in the ABCA1 expression in RAR-TAT (*P*= 0.028), RDR-RDT (*P*=0.014), and RDR-TDT (*P*=0.005). In the Aβ groups, pre-conditioning, post-conditioning, and pre-post conditioning increased the ABCA1 expression, but a significant increase was observed only between training before and after Aβ injection and resting before and after Aβ injection. In the sham groups, pre-post conditioning showed a significant increase in the ABCA1 expression compared to resting before and after the DMSO injection. Therefore, the highest increase in ABCA1 expression was observed in the groups that trained before and after the injection, and the exercise before or after the injection alone could not significantly increase the ABCA1 expression.

The results of the *post hoc* test showed a significant difference in the level of soluble Aβ1-42 in the RAR-TAT (*P*=0.000), TAR-TAT (*P*=0.000), and RAT-TAT (*P*= 0.000) groups. According to these results, the level of soluble Aβ1-42 in the TAT group was significantly lower than the other groups. Also, there was no significant difference between the groups that trained before or after the injection. These results indicated that aerobic training can reduce the level of soluble Aβ1-42 and that compared to pre- or post-conditioning, pre-post-conditioning has a greater effect on reducing the level of soluble Aβ1-42 in the hippocampus.

***Analysis of behavioral test***

To measure the cognitive function, 56 male Wistar rats participated in the behavioral test. Figures 3 shows the escape latency, the traveled distance, and the time in the target quadrant (probe trial) of male Wistar rats in the eight groups. The results of the MANOVA test showed a significant difference between the eight groups in behavioral variables: Wilks ˄ = 0.092, F (21, 132.6) = 8.178, *P*=0.000, Eta = 0.548, which indicates that aerobic training has a significant effect on the learning and memory of rats (*P*<0.01), and 55% of the variability of these three variables was influenced by aerobic training in this study. Table 4 shows the results of a one-way ANOVA for comparing the behavioral variables in rats between the eight groups.

The results of Table 4 show that aerobic training caused a significant decrease in the escape latency (*P*<0.01) and the traveled distance (*P*<0.01) and a significant increase in the time spent in the target quadrant (*P*<0.01) in the trained groups. These results indicate the effect of the training program on the cognitive function in both the sham and Aβ groups. 

The results of the *post hoc* test showed a significant difference in the escape latency, the traveled distance, and the time spent in the target quadrant between the sham and the Aβ groups. The comparison of scores between RAR-RDR, RAT-RDT, TAR-TDR, and TAT-TDT (*P*<0.01) showed that in all groups, the rats in the Aβ group showed the worse scores for all three variables. These results indicate cognitive impairment in the rats that were exposed to the induction of AD. 

The results of the *post hoc* test showed a significant difference in the escape latency, the traveled distance, and the time spent in the target quadrant between the TAT, TAR, and RAT groups with the RAR group (*P*<0.01). However, there was no significant difference between TAT, TAR, and RAT groups in the three behavioral variables (*P*>0.05). These results indicate that pre-conditioning, post-conditioning, and pre-post conditioning play an effective role in improving cognitive function in rats with AD.

In addition, the results of the *post hoc* test showed a significant difference in the escape latency, the traveled distance, and the time spent in the target quadrants between the TDT and RDT groups with the RDR group (*P*<0.01). There was no significant difference between the TDT and RDT groups (*P*>0.05), indicating no significant difference between four and eight weeks of aerobic training. Therefore, in this study, aerobic training improved the cognitive function in the rats with no AD.

## Discussion

The results of this study showed that aerobic training before and after the AD induction increased the ABCA1 expression in the hippocampus of rats in both the sham and Aβ groups. There was no significant difference in APOE expression between the eight groups. The expression of ABCA1 and APOE is regulated by nuclear receptors, the peroxisome proliferator-activated receptor γ (PPAR-γ), and the liver X receptors (LXRs) (21, 44). In the metabolic pathway, PPAR-γ results in the expression of LXR and ultimately its target genes ABCA1 and APOE (45-49). Thus, increasing the expression of the ABCA1 gene through the LXR and PPAR-γ agonists results in the synthesis of APOE-containing HDL particles. As a result, the lipidated APOE facilitates the intracellular and extracellular proteolytic degradation of Aβ by NEP and IDE and promotes the clearance of Aβ (50).

Several studies have been conducted on the role of ABCA1 and APOE in AD pathology and most of these have examined the effects of LXR and PPAR-γ agonists and genetic interventions (e.g. genetic overexpression or deletion of ABCA1). The results of these studies have shown that the effects of RXR and LXR agonists increase the expression of ABCA1 and APOE in mice and promote the clearance of sAβ and improve cognitive impairment (35-37). The genetic overexpression of ABCA1 in transgenic mice showed a decrease in the soluble Aβ and Aβ plaques (34), and the deletion of ABCA1 increases the soluble and insoluble Aβ levels and leads to the progress of the pathology of Aβ plaques in AD mice (32). Other studies in APP transgenic rats have shown that the deletion of ABCA1 leads to increased Aβ deposition and a significant decrease in the total APOE in the brain (29, 31). Therefore, in this study, aerobic exercise before and after AD induction, possibly similar to LXR and PPAR-γ agonists, increased the expression of ABCA1 in both the Aβ and the sham groups. Thus, aerobic exercise can be used as an effective alternative method to drugs and genetic interventions and will be considered in improving the pathology of AD. 

On the other hand, studies have shown that exercise can promote the clearance of Aβ and decrease the level of soluble Aβ in Alzheimer’s transgenic mice (51-53). The results of the study indicate that the clearance of Aβ increases via mechanisms like degrading enzymes (54), neural mechanisms (55), and neural growth factors (51). The results of drug and genetic interventions also show a decrease in the level of soluble Aβ along with an increase in the expression of ABCA1. The study results showed that the expression of ABCA1 increased and the level of soluble Aβ1-42 decreased in the hippocampus of rats in the training group compared to the resting group. Therefore, aerobic training before and after the induction of AD probably by increasing the expression of ABCA1 leads to a decrease in the level of soluble Aβ1-42 in the hippocampus of rats. The proposed mechanism of these changes can be explained by an increase in the level of lipidated APOE and a facilitation of the degradation of Aβ (Figure 4). The better cognitive function and the lower level of soluble Aβ1-42 in the hippocampus, coupled with an increase in the expression of ABCA1, expresses the effects of aerobic training before and after AD induction on the disease pathology.

Most studies indicate that exercise and physical activity improve cognitive function (24, 25). Research has shown the effect of physical activity on CNS adaptations, especially in the hippocampus. Exercise and physical activity lead to neurogenesis, as well as changes in the synaptic plasticity in the hippocampus, which improves cognitive functions, such as learning and memory (26, 27, 56). The results of the behavioral tests in this study showed that the rats of Aβ group had a significantly worse performance than the sham group in the memory and learning tests. These results indicated cognitive impairment and AD induction in the Aβ group. In addition, the effects of exercise in both Aβ and sham groups indicated an improvement in the cognitive function as a result of aerobic exercise. Therefore, the results of this study showed that aerobic training before and after the induction of AD, with the effect on the metabolism of lipid in the brain and increasing the expression of the ABCA1 and the clearance of Aβ1-42 in the hippocampus, could improve the cognitive function of rats.

Most studies have shown that the APOE genotype is the strongest genetic risk factor for AD (12, 57). In humans, there are three isoforms: APOE2, APOE3, and APOE4 (58). APOE4 increases the risk of AD (57, 59), while APOE2 and APOE3 are associated with reducing the risk (60). APOE2, especially, inhibits Aβ deposition and plays a protective role (14, 61). The results of research indicate that the APOE4 aﬃnity to complex with Aβ is lower than APOE2 and APOE3 (62). 

## Conclusion

APOE4 is thus considered to be the strongest risk factor for AD because APOE4 increases the Aβ deposition in amyloid plaques and inhibits the clearance of Aβ. In addition, the lipidation of APOE increases the affinity for Aβ, and any method that can increase the level of lipidated APOE can be effective in preventing and treating AD. According to the importance of ABCA1 and APOE in the pathology of AD, and according to the outcomes of this study, aerobic exercise before and after the induction of AD can be used as an alternative to genetic and pharmaceutical interventions. It can have beneficial effects on the prevention and reduction of the symptoms of AD. 
